# Enhancing the interpretation of statistical *P* values in toxicology studies: implementation of linear mixed models (LMMs) and standardized effect sizes (SESs)

**DOI:** 10.1007/s00204-015-1487-8

**Published:** 2015-02-28

**Authors:** Kerstin Schmidt, Jörg Schmidtke, Christian Kohl, Ralf Wilhelm, Joachim Schiemann, Hilko van der Voet, Pablo Steinberg

**Affiliations:** 1 BioMath GmbH, Schnickmannstr. 4, 18055 Rostock, Germany; 2 Institute for Biosafety in Plant Biotechnology, Julius Kühn-Institut, Federal Research Centre for Cultivated Plants, Erwin-Baur-Str. 27, 06484 Quedlinburg, Germany; 3 Biometris, Wageningen UR, P.O. Box 16, 6700 AA Wageningen, The Netherlands; 4 Institute for Food Toxicology and Analytical Chemistry, University of Veterinary Medicine Hannover, Bischofsholer Damm 15, 30173 Hannover, Germany

**Keywords:** *P* values, ANOVA, Linear mixed models, Standardized effect sizes, 90-day toxicity study, Repeated measurements

## Abstract

In this paper, we compare the traditional ANOVA approach to analysing data from 90-day toxicity studies with a more modern LMM approach, and we investigate the use of standardized effect sizes. The LMM approach is used to analyse weight or feed consumption data. When compared to the week-by-week ANOVA with multiple test results per week, this approach results in *only one* statement on differences in weight development between groups. Standardized effect sizes are calculated for the endpoints: weight, relative organ weights, haematology and clinical biochemistry. The endpoints are standardized, allowing *different* endpoints of the *same* study to be compared and providing an overall picture of group differences at a glance. Furthermore, in terms of standardized effect sizes, statistical significance and biological relevance are displayed simultaneously in a graph.

## Introduction

### 90-day feeding studies

OECD has developed standard procedures employing animal models to assess the toxicity of chemical compounds to humans. In this context, repeated-dose 90-day oral (subchronic) toxicity studies are usually carried out to evaluate the toxic potential of a chemical in more detail after initial information on its toxicity has been obtained from acute or repeated-dose 28-day toxicity tests. At least three dose levels of a test substance and a concurrent control are administered daily *per os* for a period of 90 days to groups of animals (OECD/OCDE 2014).

This general OECD test approach has been applied to the testing of whole food/feed derived from genetically modified organisms (GMOs) in order to consider toxic effects holistically rather than for a single compound. Toxicity studies are now a mandatory part of the risk assessment of genetically modified (GM) food and feed in Europe. Although there is a fundamental difference (dosing range) between testing chemicals and whole food/feed, repeated-dose 90-day oral toxicity studies nevertheless have been included in the integrated approach of assessing the potential toxicity of GM plants (EFSA Scientific Committee 2011). The idea is to administer diets containing the plant under study as a component: in treatment groups, this component consists of GM plant material (high and low doses), and in a control group this component consists of conventional plant material. Several observation and examination data are recorded and compared between the treatment and control groups.

In this paper, we describe the statistical methods used for analysing the data from the GRACE 90-day studies (Zeljenková et al. 2014). We compare the traditional ANOVA approach with a more modern LMM approach, and we investigate the use of standardized effect sizes as proposed by EFSA (2011).

### Statistical significance and biological relevance

There are several guidelines and publications dealing with the statistical treatment of toxicity study data (e.g. Anses 2011; EFSA Scientific Committee 2011; Festing and Altman 2002; OECD Environment, Health and Safety Publications 2012). OECD in its guidance document No. 116 mentions that there is no single approach to the statistical analysis of data and that statistical methods continue to develop so that new and modified approaches may continue to be proposed (OECD Environment, Health and Safety Publications 2012). Most of the guidelines favour a traditional approach (i.e. hypothesis testing, *P* value), which simply asks ‘Is there an effect?’, while other more recently published papers promote the reporting of effect sizes and confidence intervals and to ask ‘How much of an effect is there?’ (Ellis 2010; Nuzzo 2014).

Most importantly, biological relevance should always be preferred over statistical significance in any evidence-based decision-making. Statistical analysis is a (undoubtedly very useful) tool for extracting information from data and helping scientists blend data and background knowledge to derive scientific conclusions—no more and no less. Denoting something as statistically significant does not mean it is biologically relevant. Statistical significance is determined by the precision of the measurements, and as such is not connected to the biological relevance of observed differences. Therefore, another element has to enter the discussion if biological relevance is of prime importance, as it is for decision-making in risk management. This element is the setting of limits for relevance, called ‘equivalence limits’ (European Commission 2013) or alternatively ‘limits of concern’ (EFSA Panel on Genetically Modified Organisms 2010). Statistical measures like ‘significant’ test results and *P* values always need interpretation, when one considers what they really mean: the chance of observing data under the assumption of a null hypothesis (of no correlation or no effect); therefore, they only reflect the likelihood that the null hypothesis is true. When the UK statistician Ronald Fisher introduced the *P* value in the 1920s, he did not mean it to be a definite decision basis. However, this was the beginning of a movement towards rigorous and objective decision-making based on *P* values, statistical power, false positives and false negatives—and disregarding the biological interpretation by simply classifying results as significant or not significant (Nuzzo 2014).

The discussion on the different number of significant differences reported by Lemen et al. (2002) and Séralini et al. (2007) when analysing the same MON863 90-day feeding study very nicely demonstrates this dilemma. EFSA (2007) summarized that both studies reported significant differences for the same 25 endpoints. Moreover, Lemen et al. (2002) described a further 10 significant differences not reported by Séralini et al. (2007), while Séralini et al. (2007) pointed out a further 13 significant differences not reported by Lemen et al. (2002). Furthermore, Séralini et al. (2007) found significant differences in 40 out of 494 tests and claimed that only 25 would be expected by chance alone. Such counting only causes confusion and uncertainty. As EFSA emphasizes in its study, statistically significant differences must be evaluated with respect to their biological relevance. This is equally true for non-significant differences as it would be unacceptable if biologically relevant effects went unnoticed for the lack of statistical power. For this reason, a prospective power analysis has been made mandatory in GMO risk assessment (EFSA Scientific Committee 2011). In summary, the relevance of statistical significance is limited.

### Traditional *P* value approach versus LMM and SES

When performing 90-day toxicity feeding studies, two types of endpoints are usually analysed: weight and feed consumption are recorded weekly (‘weight and feed consumption data’). Organ weights, haematology and clinical biochemistry, as well as gross necropsy and histopathology parameters, are surveyed once at the end of the study (‘other endpoints’). All these endpoints are compared between the groups and tested with relevant baseline values to identify any test substance- and dose-dependent toxic responses.

**Fig. 1:**
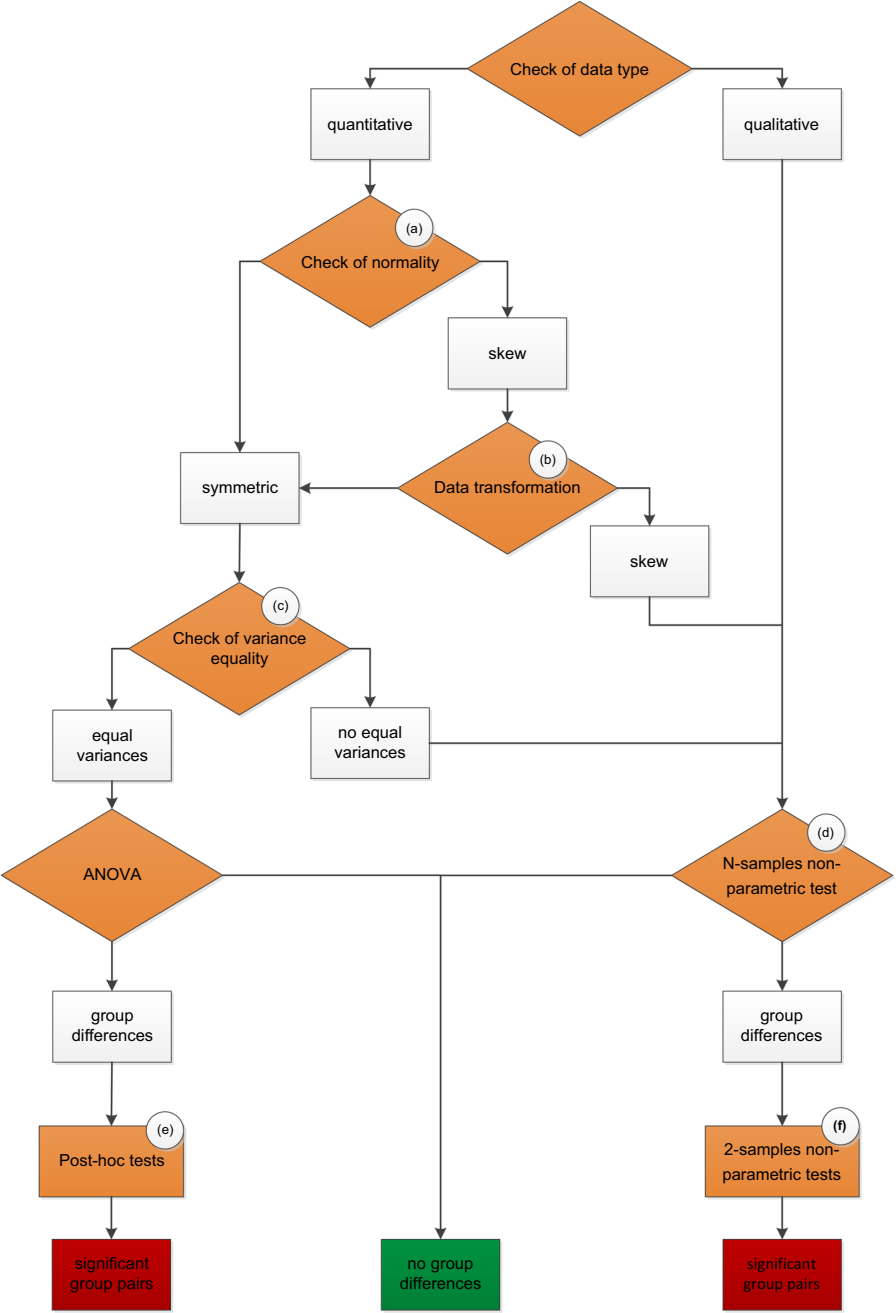
Flowchart representing a statistical decision tree for analysing data in 90-day toxicology studies. **a** Kolmogorov–Smirnov (with Lilliefors correction) test and Shapiro–Wilk test, Q–Q plots, **b** logarithmic, logit or square root transformation, **c** Levene test, **d** Kruskal–Wallis test, **e** Dunnett’s test or Turkey test, **f** Wilcoxon test

The traditional approach (i.e. hypothesis testing, *P* value) focuses on the analysis of variance (ANOVA) followed by post hoc tests. ANOVA compares group (treatments, control) means separately for each factor level (e.g. gender: male/female) and separately for each endpoint (i.e. weight data are also independently analysed week-by-week). The choice of statistical method depends on whether the data are qualitative or quantitative and whether the generic assumptions underlying the specific test are met (OECD Environment, Health and Safety Publications 2012). Figure 1 presents a typical decision tree for the choice of statistical tests when analysing toxicity studies. Following the logic of this decision tree, ANOVA is applied for quantitative data, independent observations with normally distributed residuals and with equal variances in the groups, whereas nonparametric tests are applied for qualitative data and when the assumption of normality and/or variance homogeneity are not met (according to the normality and variance homogeneity tests indicated in Fig. 1). Nonparametric tests are usually limited to these cases, since they have lower power compared to their parametric counterparts when the corresponding assumptions are met. Most of the endpoints in 90-day toxicity studies are quantitative: body and organ weights, haematology and clinical biochemistry data are continuous data, and numbers of blood cells are discrete counts. Nevertheless, the assumptions of normal distribution and variance homogeneity are often not met. In this case, data may be transformed (logarithmic, logit, square root transformation) to improve the normality or variance homogeneity. ANOVA is initially applied to test the overall hypothesis that there are no differences among the group means. In the event that ANOVA delivers a significant result, certain differences between two groups are examined case-by-case applying either post hoc tests or orthogonal contrasts. The most frequently used post hoc tests are Dunnett’s test to compare each treatment group with the control and Tukey’s test to compare groups pairwise. Gross necropsy and histopathology data are qualitative data (categorical, binary or ordinal). For qualitative data and quantitative data in which the ANOVA assumptions are not met, the Kruskal–Wallis test is applied as an overall test of significant differences, and the Wilcoxon test is applied to individually compare two groups. Note that these nonparametric tests assume equal variances as well, and in case of heteroscedasticity, the Kruskal–Wallis test is not better than an ANOVA. Nevertheless, in view of the lack of any alternative for a nonparametric test, the Kruskal–Wallis is named by the OECD Guidance Document 116 (OECD Environment, Health and Safety Publications 2012).

Since ANOVA tolerates deviations from the assumptions and parametric tests are usually more powerful and versatile, it is sometimes applied to all variables. A more conservative approach is to apply only nonparametric tests to all variables.

Finally, all results are presented in tables (group means and standard deviations per factor level) and bar or line graphs with asterisks marking significant differences.

Linear mixed models (LMMs) allow weight development in growth curves to be analysed as repeated measurements over time. They are robust with respect to the assumptions of normal distribution, homogeneity of variance and error independence. Moreover, they allow a comprehensive analysis of weight data of a study, thereby incorporating all model factors with interactions such as group, dose and gender *plus* the development over time. When compared to the week-by-week ANOVA with multiple test results (for all group comparisons) per week, this approach results in *only one* statement on differences in weight development between groups. Taking time as a fixed factor to indicate repeated measurements allows modelling of time and interactions as well as taking account of serial correlations and reducing residual variance.

An effect size in a toxicology study endpoint is the difference (e.g. treatment vs. control) of means per group. Whether the size of an effect is biologically relevant has to be assessed by comparing it to an equivalence limit or limit of concern set by a toxicologist or other expert. A standardized effect size (SES) is the difference between two group means divided by a standardizing factor, for which EFSA (2011) has proposed the pooled standard deviation (SD). With this standardization, all endpoints are transformed and expressed in SD units, allowing comparison of different endpoints (organ weights, haematology and clinical biochemistry parameters) of the same study (Festing 2014). Therefore, an overall picture of group differences is provided at a glance. Furthermore, SES enables statistical significance and biological relevance (in SD units) to be illustrated simultaneously when the equivalence limits are also indicated in the display. EFSA (2011) gives the example where differences of one unit of SD are considered of little toxicological relevance. The equivalence limits can then be set at 1 for the SES, and in this work, we will follow this example.

## Materials and methods

We used data from two 90-day feeding trials with two different GM maize MON810 varieties performed within the GRACE project (GMO Risk Assessment and Communication of Evidence; www.grace-fp7.eu) funded by the European Commission within the Seventh Framework Programme (Zeljenková et al. 2014). Both feeding trials incorporated five groups: two treatment groups (33 % GM maize [33 % GMO], 11 % GM maize [11 % GMO]), a control group (33 % control maize [control]) and two additional groups (conventional maize varieties [conventional 1] and [conventional 2]). The total number of animals per feeding trial was 160 with 16 animals (8 cages) per gender and dietary treatment.

Each animal was weighed on the first day of the feeding trial, once weekly during the feeding trial and at the end of the feeding trial. Feed consumption was determined once weekly and reported as the total amount of feed consumed by two animals in one cage per week. White blood cell count (WBC), red blood cell count (RBC), haemoglobin concentration (HGB), haematocrit (HCT), mean cell volume (MCV), mean corpuscular haemoglobin (MCH), mean corpuscular haemoglobin concentration (MCHC), platelet count (PLT), lymphocyte count (LYM) and differential leucocyte count parameters were measured for the haematology analyses. For the clinical biochemistry analyses, the parameters alkaline phosphatase (ALP), alanine aminotransferase (ALT), aspartate aminotransferase (AST), albumin (ALB), total protein (TP), glucose (GLU), creatinine (CREA), urea (U), cholesterol (CHOL), triglycerides (TRG), calcium (Ca), chloride (Cl), potassium (K), sodium (Na) and phosphorus (P) were measured. Moreover, the wet weight of the kidneys, spleen, liver, adrenal glands, pancreas, lung, heart, thymus, testes, epididymides, uterus, ovaries and brain of all animals was recorded (The collated primary data are available through the website http://www.cadima.info.).

Two animals were housed per cage. Consequently, the cage was considered the experimental unit and means per cage were calculated for all measurements prior to the statistical analysis.

### Data check and quality control

Raw data from both trials were screened for their structure and data, and variable definitions were determined. Based on these definitions, an SAS analysis data set was created. Mean values per cage (experimental unit) were calculated for all endpoints except feed consumption. Secondary variables like weight gain per week or organ/body weights were re-computed. All variables were formatted and labelled. The SAS data set was locked to exclude further modifications. An SPSS data file and an Excel file were exported from this SAS data set.

Data were screened for outliers and extreme values. Box and whisker plots were created for each gender-group factor level combination and all variables to identify extreme values (variable values within the 1.5* and 3* interquartile range and variable values outside the 3* interquartile range). Extreme values were recorded in an Excel sheet for easier identification of irregular patterns or abnormal animals. Growth curves for all animals were plotted (scatter plots, weight against study day) and visually inspected for irregular patterns.

To describe the data, summary statistics including means, standard deviations, 95 % confidence intervals, medians, number of valid values, minima and maxima were calculated and tabulated. In addition to the box and whisker plots, plots of means and 95 % confidence intervals were drawn. Descriptive analysis was performed separately for each gender and group.

To check the normality of the data, Kolmogorov–Smirnov tests (with Lilliefors correction) and Shapiro–Wilk tests were performed. When significances were identified, the corresponding normal Q–Q plots were displayed.

### Analysis of weight data

Firstly, a traditional analysis with ANOVA was carried out for weight and feed consumption data, separately for each gender and each week. For four comparisons of particular interest (control—GMO33 %, control—GMO11 %, control—conventional 1, control—conventional 2), post hoc Dunnett’s tests were performed. There were no missing data, and the data set was fully balanced in each week; therefore, the default type III sum of square procedure was used for the ANOVA. Levene’s test to check homogeneity of variance was applied. Test results were presented in tables of means and standard deviations, where all means of groups GMO11 %, GMO33 %, conventional 1 and conventional 2 differing significantly from control group means were marked with asterisks.

Secondly, weight and feed consumption data were analysed with mixed models, using the restricted maximum likelihood (REML) algorithm with Toeplitz covariance structure. Group (five levels) was considered a fixed factor. The factor week (time in weeks from the start of the experiment) or day (time in days from the start of the experiment) was considered a continuous fixed factor. For the resulting least square means, standardized effect sizes as well as their 95 % confidence intervals were calculated according to Nakagawa and Cuthill (2007).

### Analysis of all other endpoints

Firstly, a traditional frequentist analysis with ANOVA and N-sample nonparametric tests was carried out for all other endpoints separately for each gender, and post hoc Dunnett’s tests and two-sample nonparametric Wilcoxon tests were performed for four comparisons of particular interest (control—GMO33 %, control—GMO11 %, control—conventional 1, control—conventional 2).

Secondly, for all other endpoints, standardized effect sizes as well as their 95 % confidence intervals were calculated according to (Nakagawa and Cuthill 2007). The same four group pairs were compared with each other: control—GMO11 %, control—GMO33 %, control—conventional 1 and control—conventional 2. A bootstrap test was applied to compare the variability within paired sets of SES (Festing 2014). The idea of this test is to investigate whether variation among the SES in the control versus GM is greater than in the control versus conventional groups (and thus indicating that the GM food is toxic).

### Graphical presentation of all results

**Fig. 2:**
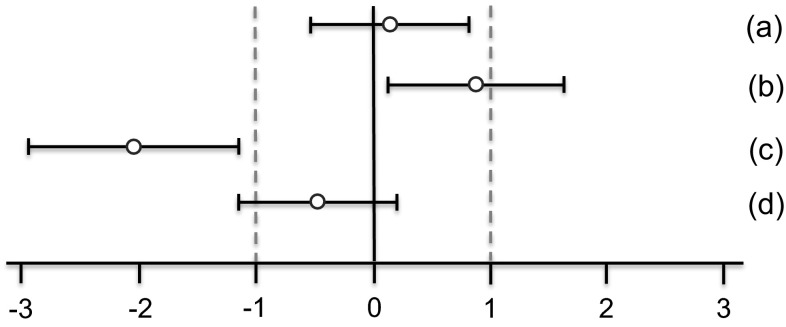
Simplified version of a graph allowing visual assessment of statistical significance and biological relevance of group comparisons. The SES point estimate (*circle*) and the 95 % confidence limits (*whiskers*, *bars* showing confidence interval) illustrate the (standardized) effect size between two groups. The *vertical black line* indicates no effect (zero difference), and *vertical grey lines* indicate biological relevance limits (here 1.0 SD, according to the study design). If the confidence interval bars cross the zero line but not the *grey lines*, therefore lie within the ±1.0 limits, there is evidence for no statistical significance as well as no biological relevance (*case a*). Two groups are significantly different when the confidence interval bars do not cross the *black vertical line* (*cases b, c*). The effect size between two groups is supposed to be biologically (toxically) relevant, when the confidence interval bars lie outside the ±1.0 limits (*case c*). *Case b* indicates statistical significance, but no clear biological relevance. *Case d* indicates no statistical significance, but no clear negation of biological relevance [reproduced from Zeljenková et al. (2014)]

All SES estimates were illustrated graphically, displaying both statistical significance and biological relevance for each of the endpoint comparison results (Fig. 2). Biological relevance here was supposed to be defined by equivalence limits of ±1.0 SD, as proposed by EFSA (2011). Body weight plus all other endpoints were shown on the same graph (separately for male and female), thereby forming an overall pattern and allowing the assessment of group comparisons at a glance.

For all analyses, we used SAS (SAS Software, version 9.4. Copyright, SAS Institute Inc. SAS and all other SAS Institute Inc. product or service names are registered trademarks or trademarks of SAS Institute Inc., Cary, NC, USA). The growth curves were also created with SAS, while the SES graphs were created with SPSS (SPSS for Windows, version 12.0. Chicago, SPSS Inc.).

## Results

In this paper, we show only the results for male rats in feeding trial B of the GRACE study (Zeljenková et al. 2014) to compare the traditional and the enhanced approach. The full statistical report by Schmidt and Schmidtke (2014) is available under www.cadima.info.

### Data quality and distribution check

The plotted growth curves did not show any irregular pattern over time. The box plot inspections identified some extreme values, mainly in the haematology and clinical biochemistry data. Most data were confirmed by the study director as not being erroneous. Two biochemistry results were excluded due to the fact that the measured values were outside the dynamic range of the analyser (animal ID 45: the potassium value, animal ID 135: the phosphorus value). No animals were excluded from the analysis.

### Weight development

**Fig. 3:**
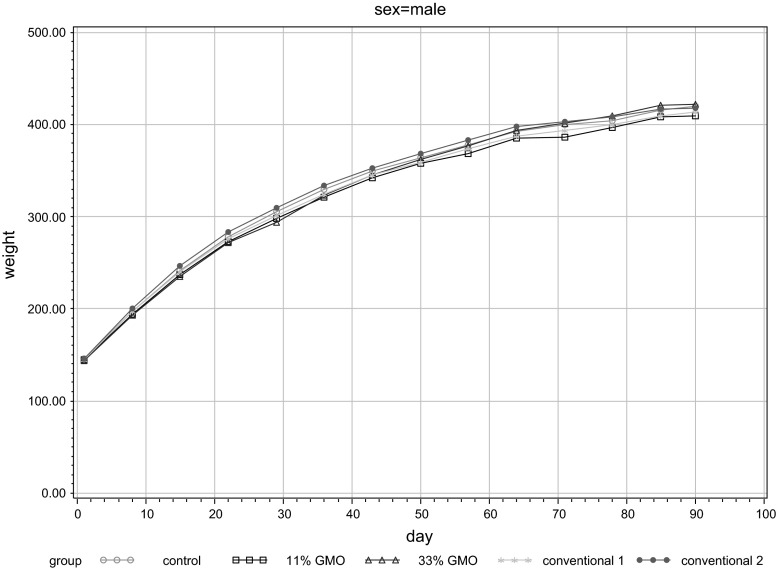
Line plot of mean male body weights (g) in feeding trial B

Levene’s test showed only few significances. The Shapiro–Wilk normality test as well as the Lillefors modification of the Kolmogorov–Smirnov test indicated only single deviations from normality. The results of ANOVA, Levene’s test and post hoc Dunnett’s test are shown in Table 1. For male rats in trial B, there were no significant differences at all. Table 2 shows the means and standard deviations for each group and each week. The weekly weight development of all groups is displayed in a line graph (Fig. 3).

**Table 1:** Test results (ANOVA, Levene’s test and post hoc Dunnett’s test) for mean male body weights (g) in feeding trial B

Endpoint	Equality of group means (ANOVA)		Homogeneity of variances: (Levene’s test)		Equality of group means post hoc tests (Dunnett)			
	*F* value	*P* value	*F* value	*P* value	Control—GMO11 %	Control—GMO33 %	Control—conventional 1	Control—conventional 2
Body weight in week 0	0.48	0.75	2.98	*0.03*	0.97	0.64	0.97	0.99
Body weight in week 1	1.18	0.34	0.61	0.66	0.85	0.60	1.00	0.83
Body weight in week 2	1.69	0.17	0.42	0.79	0.80	0.46	0.99	0.67
Body weight in week 3	1.13	0.36	0.85	0.50	0.79	0.67	0.99	0.83
Body weight in week 4	1.16	0.35	1.99	0.12	0.78	0.43	0.96	0.96
Body weight in week 5	0.74	0.57	1.48	0.23	0.75	0.85	0.92	0.96
Body weight in week 6	0.46	0.76	1.37	0.26	0.81	0.96	0.95	0.99
Body weight in week 7	0.32	0.86	1.43	0.24	0.94	0.99	0.97	0.98
Body weight in week 8	0.49	0.75	1.52	0.22	0.78	0.99	0.98	0.98
Body weight in week 9	0.33	0.85	1.56	0.21	0.92	1.00	0.98	0.98
Body weight in week 10	0.55	0.70	1.74	0.16	0.69	0.99	0.97	0.99
Body weight in week 11	0.36	0.84	1.97	0.12	0.94	0.99	0.99	0.99
Body weight in week 12	0.32	0.86	2.02	0.11	0.95	0.98	0.98	0.99
Body weight in week 13	0.29	0.88	1.85	0.14	0.85	0.99	0.97	0.99

**Table 2:** Body weights (g) in feeding trial B, male rats (mean ± standard deviation)

Endpoint	Control	11 % GMO	33 % GMO	Conventional 1	Conventional 2
Body weight in week 0	145.70 ± 3.73	144.79 ± 1.56	143.50 ± 3.33	144.74 ± 4.14	145.97 ± 5.91
Body weight in week 1	197.31 ± 6.12	194.34 ± 8.30	192.91 ± 6.21	197.20 ± 7.04	200.44 ± 9.70
Body weight in week 2	241.62 ± 7.53	237.47 ± 9.74	235.03 ± 9.96	240.48 ± 8.92	246.72 ± 11.63
Body weight in week 3	278.37 ± 9.03	272.96 ± 13.61	271.81 ± 13.05	276.24 ± 10.22	283.42 ± 14.72
Body weight in week 4	305.13 ± 11.40	297.84 ± 16.99	293.66 ± 22.24	301.07 ± 10.33	309.36 ± 16.65
Body weight in week 5	329.59 ± 13.41	321.48 ± 18.32	322.85 ± 23.12	324.18 ± 11.21	334.07 ± 17.91
Body weight in week 6	349.99 ± 14.27	342.25 ± 21.04	345.26 ± 24.52	344.97 ± 11.29	353.04 ± 15.83
Body weight in week 7	364.05 ± 17.00	357.76 ± 25.68	362.42 ± 29.30	358.95 ± 11.56	368.45 ± 18.69
Body weight in week 8	377.98 ± 17.71	368.14 ± 21.79	376.49 ± 32.15	373.63 ± 12.59	382.67 ± 19.91
Body weight in week 9	392.65 ± 19.90	384.88 ± 29.18	393.42 ± 34.91	387.59 ± 11.16	397.77 ± 22.36
Body weight in week 10	399.65 ± 19.92	386.42 ± 34.54	401.16 ± 34.09	393.36 ± 11.97	402.69 ± 19.34
Body weight in week 11	404.31 ± 20.26	396.99 ± 31.38	409.03 ± 35.24	399.66 ± 12.14	408.15 ± 17.34
Body weight in week 12	415.46 ± 22.17	408.07 ± 34.21	421.24 ± 39.45	409.53 ± 11.07	416.83 ± 18.81
Body weight in week 13^a^	419.84 ± 22.86	409.19 ± 32.42	422.44 ± 39.81	413.54 ± 11.59	417.34 ± 21.28

^a^Week 13 = 5 days

The results of the LMM analysis are shown in Table 3(a). Significant effects of intercept were expected from the model choice. Since growth rates differ over time, a significant week/day effect was also expected. There is no group effect for weight development, and neither is there an interaction between group and week/day. Table 3(b) shows the least square weight means (i.e. the mean weights over time) for the five groups. Table 3(c) shows the differences between least square means, indicating that no difference is significant.

**Table 3:** LMM results for weight in feeding trial B, male rats

(a) Type 3 tests of fixed effects				
Effect	Num DF	Den DF	*F* value	*P* value
Intercept	1	29.5	27.56	<*0.0001*
Group	4	29.5	0.95	0.4473
Day	1	16.4	1851.35	<*0.0001*
Day*group	4	16.4	1.06	0.4059

(b) Least squares means (*α* = 0.05)						
Group	Estimate	Standard error	DF	*t* value	Lower CI	Upper CI
Control	271.82	6.6855	31.3	40.66	258.19	285.45
11 % GMO	265.95	6.6855	31.3	39.78	252.32	279.58
33 % GMO	272.60	6.6855	31.3	40.78	258.98	286.23
Conventional 1	268.73	6.6855	31.3	40.20	255.10	282.36
Conventional 2	270.06	6.6855	31.3	40.40	256.43	283.69

(c) Differences of least squares means (*α* = 0.05)								
Group	Group	Estimate	Standard error	DF	*t* value	*P* value	Lower CI	Upper CI
11 % GMO	33 % GMO	−6.6580	9.4547	31.3	−0.70	0.4865	−25.9324	12.6165
11 % GMO	Control	−5.8732	9.4547	31.3	−0.62	0.5390	−25.1476	13.4012
11 % GMO	Conventional 1	−2.7831	9.4547	31.3	−0.29	0.7704	−22.0575	16.4913
11 % GMO	Conventional 2	−4.1152	9.4547	31.3	−0.44	0.6664	−23.3896	15.1592
33 % GMO	Control	0.7848	9.4547	31.3	0.08	0.9344	−18.4896	20.0592
33 % GMO	Conventional 1	3.8748	9.4547	31.3	0.41	0.6847	−15.3996	23.1493
33 % GMO	Conventional 2	2.5428	9.4547	31.3	0.27	0.7897	−16.7316	21.8172
Control	Conventional 1	3.0901	9.4547	31.3	0.33	0.7460	−16.1843	22.3645
Control	Conventional 2	1.7580	9.4547	31.3	0.19	0.8537	−17.5164	21.0324
Conventional 1	Conventional 2	−1.3320	9.4547	31.3	−0.14	0.8889	−20.6065	17.9424

(d) SES with confidence intervals for mean weight in feeding trial B, male rats			
Groups	SES	Lower CI	Upper CI
Control—11 % GMO	0.1550	−0.8993	1.2093
Control—33 % GMO	−0.0200	−1.0727	1.0327
Control—conventional 1	0.0825	−0.9706	1.1356
Control—conventional 2	0.0475	−1.0053	1.1003

The SES and confidence intervals for the least square means are shown in Table 3(d).

### Other endpoints

Results of normality and variance homogeneity testing for all other endpoints are presented in Table 4. Significant test results, indicating that data are not normally distributed or variances are not homogeneous, are italicized. Consequently, column 10 states whether parametric or nonparametric tests should be applied.

**Table 4:** Check of assumptions for parametric testing for all other endpoints (relative organ weights, haematology and clinical biochemistry parameters), trial B, male rats

Endpoint^1^	Data type	Normality test					Variance homogeneity		Test procedure
1	2	3	4	5	6	7	8	9	10
		Control	GMO11 %	GMO33 %	Conventional 1	Conventional 2	Levene *F* value	Levene *P* value	
Kidney (right)	Quantitative	0.7552	0.1743	0.6231	0.3390	0.4402	0.69	0.6018	ANOVA
Kidney (left)	Quantitative	0.9656	0.3397	0.2994	0.9832	0.2097	0.52	0.7222	ANOVA
Spleen	Quantitative	*0.0326*	0.2113	0.2707	0.8962	0.1615	0.49	0.7438	Nonparametric
Liver	Quantitative	*0.0209*	0.9416	0.4679	0.4244	*0.0006*	1.22	0.3215	Nonparametric
Adrenal gland (right)	Quantitative	0.6137	0.4156	0.6572	0.6883	0.8588	0.88	0.4877	ANOVA
Adrenal gland (left)	Quantitative	*0.0002*	*0.0046*	0.8884	0.3607	0.0848	0.86	0.4995	Nonparametric
Lung	Quantitative	0.8762	0.3988	0.8425	0.5559	0.6471	3.53	0.0160	Nonparametric
Heart	Quantitative	0.9099	*0.0209*	0.3143	0.2591	0.6659	0.84	0.5077	Nonparametric
Thymus	Quantitative	0.0893	0.6558	0.4512	0.7870	0.2967	2.30	0.0783	ANOVA
Pancreas	Quantitative	0.6980	0.7304	0.5019	0.1879	0.6566	2.30	0.0779	ANOVA
Testis (right)	Quantitative	0.1688	0.5216	0.5453	0.7915	0.7838	1.14	0.3556	ANOVA
Testis (left)	Quantitative	*0.0392*	0.9329	0.0905	0.1529	0.9197	1.36	0.2662	Nonparametric
Epididymis (right)	Quantitative	0.6166	0.5988	0.6017	0.4921	0.5336	2.08	0.1040	ANOVA
Epididymis (left)	Quantitative	0.6145	0.9547	0.1445	0.1278	0.6453	1.45	0.2389	ANOVA
Brain	Quantitative	*0.0193*	0.8181	0.5585	0.0533	0.9300	1.26	0.3060	Nonparametric
WBC (10^3^/μl)	Quantitative	*0.5079*	0.8851	0.3192	0.1852	0.2697	0.66	0.6260	ANOVA
RBC (10^6^/μl)	Quantitative	0.2019	0.2975	0.9704	0.5888	0.5073	1.29	0.2912	ANOVA
HGB (g/dl)	Quantitative	0.5016	0.0653	0.8232	0.4895	0.0566	0.61	0.6562	ANOVA
HCT (%)	Quantitative	0.2604	0.2046	0.6916	0.1038	0.4706	1.12	0.3636	ANOVA
MCV (fl)	Quantitative	0.7094	0.9135	0.8920	0.4880	0.2131	0.87	0.4899	ANOVA
MCH (pg)	Quantitative	0.8333	0.6083	0.9366	0.2303	0.2983	0.89	0.4821	ANOVA
MCHC (g/dl)	Quantitative	*0.0487*	0.4282	0.8871	0.1095	0.9858	1.86	0.1396	Nonparametric
PLT (10^3^/μl)	Quantitative	*0.7684*	0.9823	0.7197	0.6863	*0.0147*	0.49	0.7448	Nonparametric
LYM (10^3^/µl)	Quantitative	0.5883	0.9267	0.7029	0.2545	*0.3944*	0.57	0.6827	ANOVA
Lymphocytes (%)	Quantitative	0.6156	*0.1883*	0.3702	0.3123	0.4707	0.53	0.7132	ANOVA
Neutrophils (%)	Quantitative	0.1503	0.1117	0.0886	0.2490	0.5507	0.92	0.4625	ANOVA
Monocytes (%)	Quantitative	0.1125	0.6722	0.9932	0.4543	*0.0464*	0.94	0.4549	Nonparametric
Eosinophils (%)	Quantitative	0.0553	*0.0337*	0.1256	0.0774	*0.0394*	3.22	*0.0238*	Nonparametric
ALP (µkat/l)	Quantitative	0.3990	0.4021^a^	0.0828	0.4416	0.0918	1.77	0.1564	ANOVA
ALT (µkat/l)	Quantitative	0.6698	0.3181^a^	0.8833	0.4495	*0.0214*	1.86	0.1387	Nonparametric
AST (µkat/l)	Quantitative	0.1471	0.0526^a^	0.6303	*0.0237*	*0.0107*	0.67	0.6155	Nonparametric
ALB (g/l)	Quantitative	0.9494	0.9656^a^	0.4145	*0.5846*	*0.6176*	1.50	0.2237	ANOVA
GLU (mmol/l)	Quantitative	*0.0386*	0.1717^a^	0.4791	0.2247	0.3033	0.34	0.8517	Nonparametric
CREA (µmol/l)	Quantitative	*0.0229*	0.5452^a^	0.8758	0.7749	0.4668	1.06	0.3896	Nonparametric
TP (g/l)	Quantitative	*0.4951*	0.8756^a^	0.0777	0.4226	0.4421	3.38	*0.0195*	Nonparametric
U (mmol/l)	Quantitative	0.8122	0.4579^a^	0.6360	0.2309	*0.0323*	2.82	*0.0397*	Nonparametric
CHOL (mmol/l)	Quantitative	0.9793	0.8580^a^	0.9743	*0.0311*	*0.2205*	0.90	*0.4747*	Nonparametric
Ca (mmol/l)	Quantitative	0.3435	0.8085^a^	0.0561	*0.0857*	0.3541	1.82	0.1479	ANOVA
Cl (mmol/l)	Quantitative	0.0516	0.1878^a^	*0.0141*	0.7365	0.4255	4.96	*0.0028*	Nonparametric
K (mmol/l)	Quantitative	0.5705	0.2488^a^	*0.6937*	0.6556	0.8520	1.52	*0.2176*	ANOVA
Na (mmol/l)	Quantitative	0.2686	0.3064^a^	0.9693	0.2321	0.5679	1.80	0.1509	ANOVA
P (mmol/l)	Quantitative	0.4402	*0.0490* ^a^	0.5375	0.5934	*0.0008*	1.46	0.2358	Nonparametric
TRG (mmol/l)	Quantitative	0.0761	*0.0103* ^a^	0.6095	0.2000	*0.9338*	1.27	0.3020	Nonparametric

*ALP* alkaline phosphatase, *ALT* alanine aminotransferase, *AST* aspartate aminotransferase, *ALB* albumin, *TP* total protein, *GLU* glucose, *CREA* creatinine, *U* urea, *CHOL* cholesterol, *TRG* triglycerides, *Ca* calcium, *Cl* chloride, *K* potassium, *Na* sodium, *P* phosphorus. Except where indicated (^a^ *n* = 15), the number of rats analysed was 16

^1^ *WBC* white blood cells, *RBC* red blood cells, *HGB* haemoglobin, *HCT* haematocrit, *MCV* mean cell volume, *MCH* mean corpuscular haemoglobin, *MCHC* mean corpuscular haemoglobin concentration, *PLT* platelets, *LYM* lymphocytes. The number of rats analysed was 16

The results of ANOVA and N-sample nonparametric tests for all other endpoints are shown in Table 5. Significant test results are italicized. Column 3 lists the ANOVA test results (*P* values) for the overall test hypothesis that there are no differences between the five groups. Column 9 includes the test results (*P* values) of the nonparametric counterpart (Kruskal–Wallis test). Columns 4–7 show the Dunnett’s test results (*P* values) post hoc to ANOVA for the four pairwise group comparisons of interest, while columns 9–12 show the corresponding nonparametric test results (Wilcoxon test post hoc to Kruskal–Wallis).

**Table 5:** Test results (ANOVA and post hoc *t* Dunnett’s test or Kruskal–Wallis and Wilcoxon tests) for all other endpoints, trial B, male rats

Endpoint	ANOVA		Post hoc test (Dunnett)				Kruskal–Wallis test		Wilcoxon test			
1	2	3	4	5	6	7	8	9	10	11	12	12
	*F* value	*P* value	Control—GMO11 %	Control—GMO33 %	Control—conventional 1	Control—conventional 2	*C* value	*P* value	Control—GMO11 %	Control—GMO33 %	Control—conventional 1	Control—conventional 2
Kidney (right)	0.73	0.5800	0.9999	0.7057	0.9742	0.9234	2.7274	0.6044	0.9581	0.3720	0.4309	0.8748
Kidney (left)	0.94	0.4531	0.6300	0.7907	0.9979	0.5139	4.403	0.3542	0.2701	0.2701	0.7132	0.2271
Spleen	0.45	0.7726	0.9998	0.9914	0.8345	0.6274	2.9213	0.5711	0.8748	0.4948	0.4309	0.1563
Liver	0.24	0.9115	0.9799	1	0.8174	0.9988	2.5189	0.6413	0.7132	0.4309	0.9581	0.7929
Adrenal gland (right)	1.44	0.2415	0.9168	0.9990	0.3004	0.9239	5.4293	0.2460	0.9581	0.9581	0.1278	0.5635
Adrenal gland (left)	0.14	0.9650	1	0.9977	0.9863	0.9896	1.9482	0.7453	0.9581	0.1278	0.4309	1
Lung	2.86	*0.0378*	*0.0428*	0.4243	0.8308	*0.0274*	12.0201	*0.0172*	*0.0039*	0.2271	0.1563	*0.0136*
Heart	0.93	0.4576	0.4022	0.5092	1	0.9330	3.889	0.4212	0.1036	0.3184	0.9581	0.7132
Thymus	1.54	0.2123	1	0.3042	0.3294	0.4088	5.3689	0.2515	1	0.0661	0.2701	0.0661
Pancreas	1.25	0.3093	0.4002	0.2219	0.9545	0.3056	4.7049	0.3189	0.1893	0.1563	0.5635	0.1893
Testis (right)	0.88	0.4848	0.4329	0.9977	0.9473	1	2.6652	0.6153	0.2701	0.9581	0.4948	0.6365
Testis (left)	1.64	0.1873	0.1834	0.9985	1	0.9999	4.4104	0.3533	0.1893	0.4948	0.5635	0.9581
Epididymis (right)	1.01	0.4132	0.2981	0.2622	0.8251	0.8183	3.2799	0.5121	0.1278	0.2701	0.3720	0.2701
Epididymis (left)	1.02	0.4098	0.3797	0.9999	0.9985	0.6612	3.2433	0.5180	0.2701	0.8748	0.6365	0.2271
Brain	0.72	0.5854	0.4038	0.9974	0.9982	0.8008	4.1067	0.3918	0.1563	0.5635	0.7132	0.2701
WBC (10^3^/μl)	3.65	*0.0137*	0.6796	0.0577	0.9470	*0.0097*	12.4125	*0.0145*	0.2271	*0.0136*	0.7132	*0.0101*
RBC (10^6^/μl)	3.76	*0.0120*	*0.0401*	0.0745	0.9981	0.9866	11.8354	*0.0186*	*0.0312*	*0.0312*	0.6740	0.5280
HGB (g/dl)	0.62	0.6544	0.5044	0.7510	0.9997	0.9888	3.2255	0.5208	0.0806	0.4613	0.9580	0.3667
HCT (%)	3.29	*0.0216*	*0.0480*	0.1132	0.9988	0.9679	12.4105	*0.0145*	*0.0101*	0.0661	0.9581	0.4309
MCV (fl)	0.27	0.8956	0.9679	0.8858	1	0.9998	1.5809	0.8122	0.6355	0.4299	0.9162	0.7130
MCH (pg)	2.51	0.0593	0.2189	0.2092	0.9630	1	8.1746	0.0854	0.0740	0.0740	0.3181	0.7132
MCHC (g/dl)	5.84	*0.0010*	*0.0187*	*0.0279*	0.8490	0.9867	14.3014	*0.0064*	*0.0306*	0.0738	0.2069	0.6358
PLT (10^3^/μl)	2.25	0.0833	0.9990	0.8543	0.5979	0.0389	8.3415	0.0798	0.6365	0.5635	0.2701	*0.0181*
LYM (10^3^/µl)	3.71	*0.0127*	0.8909	0.0906	0.9975	*0.0130*	11.8671	*0.0184*	0.4309	*0.0313*	1	*0.0101*
Lymphocytes (%)	0.5	0.7377	0.9872	0.5068	0.9657	0.7875	2.7915	0.5933	0.3713	0.0740	0.4302	0.4295
Neutrophils (%)	0.18	0.9477	0.9584	0.9931	1	1	0.4828	0.9752	1	0.4613	0.7126	0.8747
Monocytes (%)	5.33	*0.0018*	*0.0017*	*0.0167*	0.0888	*0.0011*	13.0694	*0.0109*	*0.0071*	*0.0307*	*0.0385*	*0.0052*
Eosinophils (%)	0.73	0.5163	0.8042	0.9159	0.9891	0.8835	3.3323	0.5838	0.4240	0.4229	0.9149	0.3138
ALP (µkat/l)	2.63	0.0510	0.9106	0.1682	0.9890	0.5801	9.8671	*0.0427*	0.7929	0.2271	1	0.2268
ALT (µkat/l)	1.04	0.4028	0.9949	1	0.9421	0.3666	2.1979	0.6994	0.7924	0.8747	0.2247	0.6358
AST (µkat/l)	0.46	0.7660	0.9207	0.7470	0.9998	0.9985	3.0909	0.5427	0.3439	0.4309	0.9163	0.7924
ALB (g/l)	0.95	0.4490	0.9943	0.9811	1	0.4117	2.8383	0.5852	0.9581	0.8748	1	0.1893
GLU (mmol/l)	1.05	0.3936	0.9976	0.9982	0.5170	0.7057	4.7488	0.3141	0.8748	0.7132	0.1893	0.2271
CREA (µmol/l)	2.39	0.0692	0.9990	1	0.0536	0.5405	8.7794	0.0669	0.2933	0.4948	*0.0406*	0.1036
TP (g/l)	0.11	0.9779	0.9995	0.9765	0.9968	0.9985	0.7705	0.9424	0.7132	0.7927	0.4948	1
U (mmol/l)	2.18	0.0917	0.3558	0.1073	0.1152	*0.0374*	8.4547	0.0763	*0.0312*	*0.0181*	*0.0458*	*0.0406*
CHOL (mmol/l)	2.17	0.0929	0.9939	0.5368	0.4232	0.7134	6.636	0.1564	0.7927	0.3184	0.3717	0.3184
Ca (mmol/l)	6.61	*0.0004*	*0.0019*	*0.0033*	0.9988	0.2341	20.0507	*0.0005*	*0.0180*	*0.0019*	0.7927	0.1559
Cl (mmol/l)	0.99	0.4264	0.4726	0.7421	0.6520	0.1847	5.3122	0.2567	0.3706	0.0508	0.1873	*0.0395*
K (mmol/l)	0.75	0.5635	0.9995	0.7256	0.9372	0.3796	3.3948	0.4941	0.8744	0.1028	0.6350	0.2254
Na (mmol/l)	2.85	*0.0383*	*0.0141*	0.2325	0.9548	0.4501	9.784	*0.0442*	*0.0132*	*0.0444*	0.5932	0.3703
P (mmol/l)	1.91	0.1301	0.3984	0.9369	0.9999	0.1169	14.807	*0.0051*	0.0019	0.2701	0.5635	0.0657
TRG (mmol/l)	1.61	0.1943	0.4719	0.7822	0.8208	0.6772	9.6928	*0.0459*	0.2271	0.1893	0.7132	0.1563

*ALP* alkaline phosphatase, *ALT* alanine aminotransferase, *AST* aspartate aminotransferase, *ALB* albumin, *TP* total protein, *GLU* glucose, *CREA* creatinine, *U* urea, *CHOL* cholesterol, *TRG* triglycerides, *Ca* calcium, *Cl* chloride, *K* potassium, *Na* sodium, *P* phosphorus

^1^ *WBC* white blood cells, *RBC* red blood cells, *HGB* haemoglobin, *HCT* haematocrit, *MCV* mean cell volume, *MCH* mean corpuscular haemoglobin, *MCHC* mean corpuscular haemoglobin concentration, *PLT* platelets, *LYM* lymphocytes. The number of rats analysed was 16

As is usual, all test results are presented in the form of tables with means and standard deviations for each group, and significant differences are marked with asterisks (Table 6).

**Table 6:** Relative organ weights, haematology and clinical biochemistry parameters (cage mean ± SD) of male rats in the feeding trial B

Endpoint^1^	Control	11 % GMO	33 % GMO	Conventional 1	Conventional 2
1	2	3	4	5	6
Kidney (right)	0.293 ± 0.019	0.292 ± 0.015	0.285 ± 0.019	0.289 ± 0.011	0.298 ± 0.015
Kidney (left)	0.283 ± 0.019	0.293 ± 0.014	0.291 ± 0.020	0.281 ± 0.014	0.294 ± 0.017
Spleen	0.197 ± 0.014	0.196 ± 0.013	0.194 ± 0.020	0.190 ± 0.015	0.187 ± 0.017
Liver	2.305 ± 0.294	2.267 ± 0.088	2.304 ± 0.093	2.230 ± 0.102	2.287 ± 0.215
Adrenal gland (right)	0.006 ± 0.001	0.006 ± 0.001	0.006 ± 0.001	0.005 ± 0.001	0.006 ± 0.001
Adrenal gland (left)	0.007 ± 0.002	0.007 ± 0.002	0.007 ± 0.001	0.007 ± 0.001	0.007 ± 0.001
Lung	0.304 ± 0.016	*0.340* ± 0.020*	0.324 ± 0.046	0.315 ± 0.016	*0.343* ± 0.030*
Heart	0.225 ± 0.010	0.233 ± 0.013	0.232 ± 0.013	0.225 ± 0.006	0.228 ± 0.012
Thymus	0.120 ± 0.018	0.121 ± 0.023	0.105 ± 0.013	0.105 ± 0.025	0.107 ± 0.010
Pancreas	0.141 ± 0.026	0.129 ± 0.011	0.126 ± 0.013	0.137 ± 0.016	0.127 ± 0.013
Testis (right)	0.472 ± 0.039	0.501 ± 0.057	0.468 ± 0.032	0.484 ± 0.046	0.473 ± 0.022
Testis (left)	0.475 ± 0.038	0.514 ± 0.059	0.471 ± 0.043	0.475 ± 0.024	0.473 ± 0.027
Epididymis (right)	0.147 ± 0.012	0.159 ± 0.016	0.160 ± 0.024	0.153 ± 0.009	0.153 ± 0.007
Epididymis (left)	0.151 ± 0.013	0.162 ± 0.018	0.150 ± 0.020	0.153 ± 0.009	0.159 ± 0.007
Brain	0.522 ± 0.027	0.545 ± 0.035	0.526 ± 0.045	0.526 ± 0.015	0.536 ± 0.029
WBC (103/μl)	9.44 ± 1.67	10.57 ± 1.53	*12.12* ± 1.95*	10.04 ± 2.60	*12.91* ± 2.70*
RBC (106/μl)	8.50 ± 0.23	*8.86* ± 0.29*	8.82 ± 0.37	8.46 ± 0.15	8.55 ± 0.30
HGB (g/dl)	16.39 ± 0.32	16.69 ± 0.45	16.61 ± 0.55	16.43 ± 0.49	16.48 ± 0.41
HCT (%)	47.21 ± 1.09	*49.04* ± 1.41*	48.76 ± 2.00	47.06 ± 1.21	47.55 ± 1.22
MCV (fl)	55.60 ± 0.73	55.38 ± 1.22	55.27 ± 0.82	55.61 ± 0.78	55.66 ± 1.02
MCH (pg)	19.31 ± 0.39	18.85 ± 0.63	18.84 ± 0.59	19.43 ± 0.46	19.30 ± 0.35
MCHC (g/dl)	34.73 ± 0.38	*34.04* ± 0.53*	*34.08* ± 0.66*	34.91 ± 0.43	34.64 ± 0.17
PLT (103/μl)	838.13 ± 60.16	844.06 ± 68.74	862.19 ± 74.94	874.44 ± 51.34	*921.13* ± 52.71*
LYM (103/μl)	8.17 ± 1.38	8.69 ± 1.32	*9.88* ± 1.50*	8.35 ± 1.95	10.50 ± 1.23 *
Lymphocytes (%)	78.66 ± 2.44	79.25 ± 3.75	80.72 ± 2.26	79.44 ± 3.50	80.06 ± 3.60
Neutrophils (%)	14.91 ± 1.81	15.63 ± 3.79	14.47 ± 1.88	15.00 ± 3.32	14.97 ± 2.43
Monocytes (%)	4.81 ± 0.98	*3.13* ± 0.64*	*3.50* ± 1.13*	*3.81* ± 0.73*	*3.06* ± 0.78*
Eosinophils (%)	1.59 ± 0.72	1.97 ± 0.99	1.31 ± 0.65	1.75 ± 1.15	1.91 ± 0.76
ALP (μkat/l)	1.34 ± 0.20	1.39 ± 0.13^a^	1.50 ± 0.17	1.37 ± 0.20	1.24 ± 0.08
ALT (μkat/l)	0.61 ± 0.05	0.60 ± 0.07^a^	0.61 ± 0.04	0.64 ± 0.04	0.69 ± 0.20
AST(μkat/l)	0.96 ± 0.16	1.00 ± 0.08^a^	1.02 ± 0.14	0.97 ± 0.14	0.94 ± 0.13
ALB (g/l)	33.61 ± 1.07	33.85 ± 1.72^a^	33.27 ± 1.70	33.63 ± 0.73	32.43 ± 2.37
GLU (mmol/l)	9.41 ± 1.72	9.21 ± 1.32^a^	9.22 ± 1.95	10.52 ± 1.95	10.28 ± 1.58
CREA (μmol/l)	41.19 ± 6.92	41.68 ± 3.10^a^	41.28 ± 3.10	*47.68* ± 6.46*	44.40 ± 4.82
TP (g/l)	59.59 ± 1.34	59.83 ± 3.66^a^	58.93 ± 4.28	59.20 ± 2.25	59.27 ± 2.49
U (mmol/l)	5.62 ± 0.42	*6.20* ± *0.47* ^a^*	*6.45* ± 0.67*	*6.43* ± 0.78*	*6.63* ± 1.18*
CHOL (mmol/l)	2.30 ± 0.24	2.26 ± 0.24^a^	2.45 ± 0.32	2.47 ± 0.24	2.17 ± 0.18
TRG (mmol/l)	0.65 ± 0.37^a^	0.84 ± 0.40^a^	0.78 ± 0.22	0.54 ± 0.16	0.80 ± 0.14
Ca (mmol/l)	2.40 ± 0.22^a^	*2.75* ± *0.23* ^a^*	*2.73* ± 0.07*	2.42 ± 0.17	2.56 ± 0.18
Cl (mmol/l)	109.81 ± 1.67	107.25 ± 6.24^a^	108.00 ± 3.75	107.75 ± 2.75	*106.16* ± 2.89*
K (mmol/l)	4.47 ± 0.14^a^	4.50 ± 0.43^a^	4.66 ± 0.24	4.58 ± 0.43	4.76 ± 0.53
Na (mmol/l)	149.94 ± 0.78^a^	*144.53* ± *5.40* ^a^*	*146.77* ± 3.07*	149.00 ± 2.30	147.50 ± 4.15
P (mmol/l)	2.51 ± 0.16^a^	*2.84* ± *0.19* ^a^*	2.64 ± 0.22	2.48 ± 0.12	2.99 ± 0.95

*ALP* alkaline phosphatase, *ALT* alanine aminotransferase, *AST* aspartate aminotransferase, *ALB* albumin, *TP* total protein, *GLU* glucose, *CREA* creatinine, *U* urea, *CHOL* cholesterol, *TRG* triglycerides, *Ca* calcium, *Cl* chloride, *K* potassium, *Na* sodium, *P* phosphorus. Except where indicated (^a^ *n* = 15), the number of rats analysed was 16

* Statistically significant difference to control group (*P* < 0.05) based on one-way ANOVA and post hoc Dunnett’s test or Kruskal–Wallis and Wilcoxon tests

^1^ *WBC* white blood cells, *RBC* red blood cells, *HGB* haemoglobin, *HCT* haematocrit, *MCV* mean cell volume, *MCH* mean corpuscular haemoglobin, *MCHC* mean corpuscular haemoglobin concentration, *PLT* platelets, *LYM* lymphocytes. The number of rats analysed was 16

Standardized effects sizes (SES) and confidence intervals are shown in Table 7. Confidence intervals not including the zero value, therefore indicating a significant difference between the groups, are italicized.

**Table 7:** SES with confidence intervals for other variables in feeding trial B, male rats

Endpoint 1	SES and confidence intervals											
	Control—GMO11 % 2			Control—GMO33 % 3			Control—conventional 1 4			Control—conventional 2 5		
	SES	Lower confidence limit	Upper confidence limit	SES	Lower confidence limit	Upper confidence limit	SES	Lower confidence limit	Upper confidence limit	SES	Lower confidence limit	Upper confidence limit
Kidney (right)	−0.05081	−1.1037	1.0021	−0.42841	−1.4931	0.6363	−0.23936	−1.2958	0.8171	0.29442	−0.7640	1.3528
Kidney (left)	0.57251	−0.5015	1.6466	0.38151	−0.6807	1.4437	−0.11841	−1.1720	0.9352	0.61097	−0.4660	1.6879
Spleen	−0.07764	−1.1307	0.9754	−0.15657	−1.2109	0.8977	−0.44094	−1.5063	0.6245	−0.57193	−1.6459	0.5021
Liver	−0.17435	−1.2290	0.8803	−0.00474	−1.0574	1.0480	−0.34006	−1.4003	0.7202	−0.06940	−1.1224	0.9836
Adrenal gland (right)	−0.27683	−1.3346	0.7809	−0.09351	−1.1468	0.9598	−0.91652	−2.0231	0.1901	0.30400	−0.7548	1.3628
Adrenal gland (left)	0.01148	−1.0412	1.0642	0.09983	−0.9535	1.1532	−0.16745	−1.2220	0.8871	−0.15182	−1.2060	0.9024
Lung	*2.01560*	*0.7230*	*3.3082*	0.58474	−0.4902	1.6597	0.71950	−0.3667	1.8057	*1.64501*	*0.4272*	*2.8628*
Heart	0.69779	−0.3865	1.7820	0.61135	−0.4657	1.6884	0.03597	−1.0168	1.0887	0.30600	−0.7528	1.3648
Thymus	0.02758	−1.0252	1.0803	−0.97323	−2.0865	0.1400	−0.68434	−1.7674	0.3987	−0.94684	−2.0569	0.1633
Pancreas	−0.60751	−1.6842	0.4692	−0.73997	−1.8281	0.3482	−0.20526	−1.2607	0.8502	−0.66540	−1.7468	0.4160
Testis (right)	0.59153	−0.4839	1.6670	−0.13638	−1.1903	0.9175	0.26809	−0.7893	1.3255	0.00645	−1.0462	1.0591
Testis (left)	0.78392	−0.3085	1.8763	−0.10546	−1.1589	0.9480	0.00318	−1.0495	1.0559	−0.06413	−1.1171	0.9888
Epididymis (right)	0.85988	−0.2404	1.9601	0.68238	−0.4005	1.7653	0.56780	−0.5059	1.6415	0.62627	−0.4519	1.7045
Epididymis (left)	0.68394	−0.3991	1.7670	−0.04811	−1.1010	1.0047	0.13796	−0.9160	1.1919	0.72817	−0.3588	1.8152
Brain	0.75119	−0.3380	1.8404	0.10418	−0.9492	1.1576	0.16403	−0.8904	1.2185	0.49081	−0.5776	1.5592
WBC (10^3^/μl)	0.70286	−0.3818	1.7876	*1.47387*	*0.2868*	*2.6609*	0.27482	−0.7828	1.3325	*1.54151*	*0.3426*	*2.7404*
RBC (10^6^/μl)	*1.40061*	*0.2259*	*2.5753*	1.06840	−0.0569	2.1937	−0.16187	−1.2163	0.8925	0.19815	−0.8571	1.2534
HGB (g/dl)	0.75829	−0.3316	1.8482	0.47522	−0.5922	1.5427	0.07505	−0.9780	1.1281	0.21985	−0.8360	1.2757
HCT (%)	*1.45208*	*0.2688*	*2.6354*	0.96313	−0.1489	2.0752	−0.12471	−1.1784	0.9290	0.29654	−0.7619	1.3550
MCV (fl)	−0.22388	−1.2799	0.8321	−0.42816	−1.4928	0.6365	0.00830	−1.0444	1.0610	0.07035	−0.9827	1.1234
MCH (pg)	−0.86979	−1.9711	0.2316	−0.92245	−2.0297	0.1848	0.29023	−0.7680	1.3485	−0.01685	−1.0696	1.0359
MCHC (g/dl)	−*1.48843*	−*2.6780*	−*0.2989*	−*1.20860*	−*2.3534*	−*0.0638*	0.45127	−0.6147	1.5173	−0.30115	−1.3598	0.7575
PLT (10^3^/μl)	0.09192	−0.9613	1.1452	0.35410	−0.7068	1.4150	0.64931	−0.4308	1.7294	*1.46750*	*0.2816*	*2.6534*
LYM (10^3^/µl)	0.38823	−0.6743	1.4508	*1.18549*	*0.0441*	*2.3269*	0.10736	−0.9461	1.1608	*1.78463*	*0.5399*	*3.0294*
Lymphocytes (%)	0.18760	−0.8674	1.2426	0.87705	−0.2251	1.9792	0.25917	−0.7979	1.3163	0.45782	−0.6086	1.5242
Neutrophils (%)	0.24188	−0.8147	1.2984	−0.23709	−1.2935	0.8193	0.03510	−1.0177	1.0879	0.02922	−1.0235	1.0820
Monocytes (%)	−*2.03851*	−*3.3361*	−*0.7409*	−*1.23867*	−*2.3879*	−*0.0895*	−*1.15815*	−*2.2957*	−*0.0206*	−*1.97990*	−*3.2649*	−*0.6949*
Eosinophils (%)	0.43463	−0.6304	1.4997	−0.41006	−1.4738	0.6536	0.16298	−0.8915	1.2174	0.42388	−0.6406	1.4883
ALP (µkat/l)	0.31500	−0.7442	1.3742	0.86780	−0.2333	1.9689	0.14515	−0.9089	1.1992	−0.63843	−1.7176	0.4407
ALT (µkat/l)	−0.23612	−1.2925	0.8202	0.04178	−1.0110	1.0946	0.60947	−0.4674	1.6863	0.53009	−0.5409	1.6011
AST (µkat/l)	0.33500	−0.7250	1.3951	0.42273	−0.6417	1.4871	0.05776	−0.9952	1.1107	−0.09771	−1.1510	0.9556
ALB (g/l)	0.17013	−0.8845	1.2247	−0.23773	−1.2941	0.8187	0.02046	−1.0323	1.0732	−0.63931	−1.7186	0.4399
GLU (mmol/l)	−0.13464	−1.1885	0.9192	−0.10404	−1.1574	0.9494	0.60102	−0.4752	1.6772	0.52667	−0.5441	1.5975
CREA (µmol/l)	0.09096	−0.9623	1.1442	0.01633	−1.0364	1.0690	0.97031	−0.1426	2.0832	0.53883	−0.5328	1.6105
TP (g/l)	0.08847	−0.9647	1.1417	−0.20868	−1.2642	0.8469	−0.20896	−1.2645	0.8466	−0.15923	−1.2136	0.8951
U (mmol/l)	*1.30292*	*0.1439*	*2.4619*	*1.48423*	*0.2954*	*2.6731*	*1.31080*	*0.1506*	*2.4710*	*1.14391*	*0.0084*	*2.2794*
CHOL (mmol/l)	−0.15872	−1.2131	0.8956	0.54714	−0.5251	1.6193	0.73248	−0.3549	1.8199	−0.57886	−1.6534	0.4956
Ca (mmol/l)	*1.56689*	*0.3635*	*2.7703*	*2.04856*	*0.7488*	*3.3484*	0.09193	−0.9613	1.1452	0.82061	−0.2755	1.9167
Cl (mmol/l)	−0.56065	−1.6338	0.5125	−0.62439	−1.7024	0.4536	−0.90653	−2.0120	0.1989	−*1.55064*	−*2.7511*	−*0.3501*
K (mmol/l)	0.09848	−0.9549	1.1518	0.94438	−0.1654	2.0542	0.35086	−0.7099	1.4116	0.73762	−0.3503	1.8255
Na (mmol/l)	−*1.40396*	−*2.5792*	−*0.2287*	−*1.41294*	−*2.5897*	−*0.2362*	−0.54637	−1.6185	0.5258	−0.81625	−1.9119	0.2794
P (mmol/l)	*1.88847*	*0.6227*	*3.1542*	0.70063	−0.3839	1.7851	−0.17744	−1.2322	0.8773	0.72102	−0.3653	1.8074
TRG (mmol/l)	0.49215	−0.5764	1.5607	0.41122	−0.6525	1.4750	−0.40973	−1.4734	0.6539	0.53012	−0.5409	1.6011

Results of ANOVA/Dunnett’s and Kruskal–Wallis/Wilcoxon test, respectively, can be directly compared with SES and their confidence intervals aligning Tables 6 and 7.

It is obvious that the patterns created by highlighting significances in italics are the same and that both approaches identified the same significances.

The results of the bootstrap test (Table 8) indicate that there are no overall differences between the groups for all comparisons of interest; i.e. that variation among the SESs does not differ between the control versus GM and the control versus conventional groups.

**Table 8:** Bootstrap test: means and confidence intervals of SES vector differences

SES difference	*N*	Mean	STD	CI95LOW	CI95UPP
DIFF_ES_21_31	58	0.04116	0.48269	−1.35008	1.43239
DIFF_ES_21_41	58	0.21315	0.58123	−1.31349	1.73979
DIFF_ES_21_51	58	−0.00969	0.57693	−1.53068	1.51130
DIFF_ES_31_41	58	0.17199	0.52662	−1.28116	1.62515
DIFF_ES_31_51	58	−0.05085	0.50284	−1.47082	1.36912

21: GMO11 %—control

31: GMO33 %—control

41: conventional 1—control

51: conventional 2—control

### Graphical presentation of all results

**Fig. 4:**
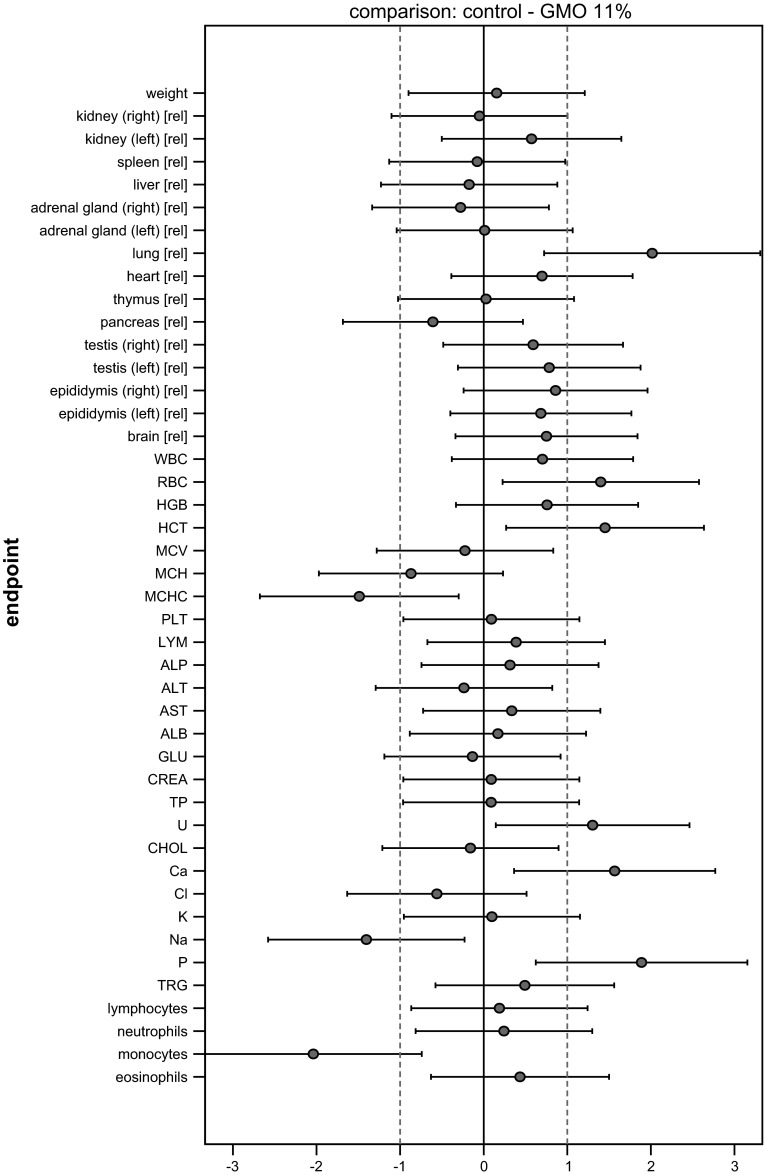

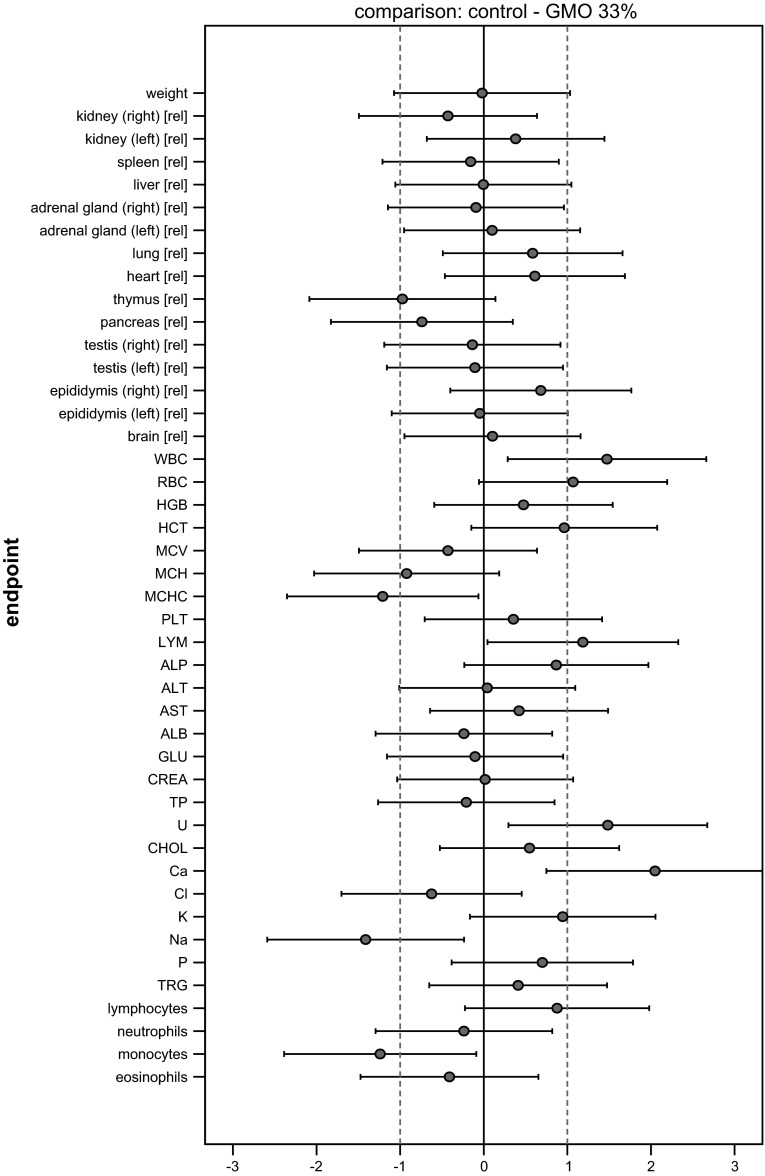

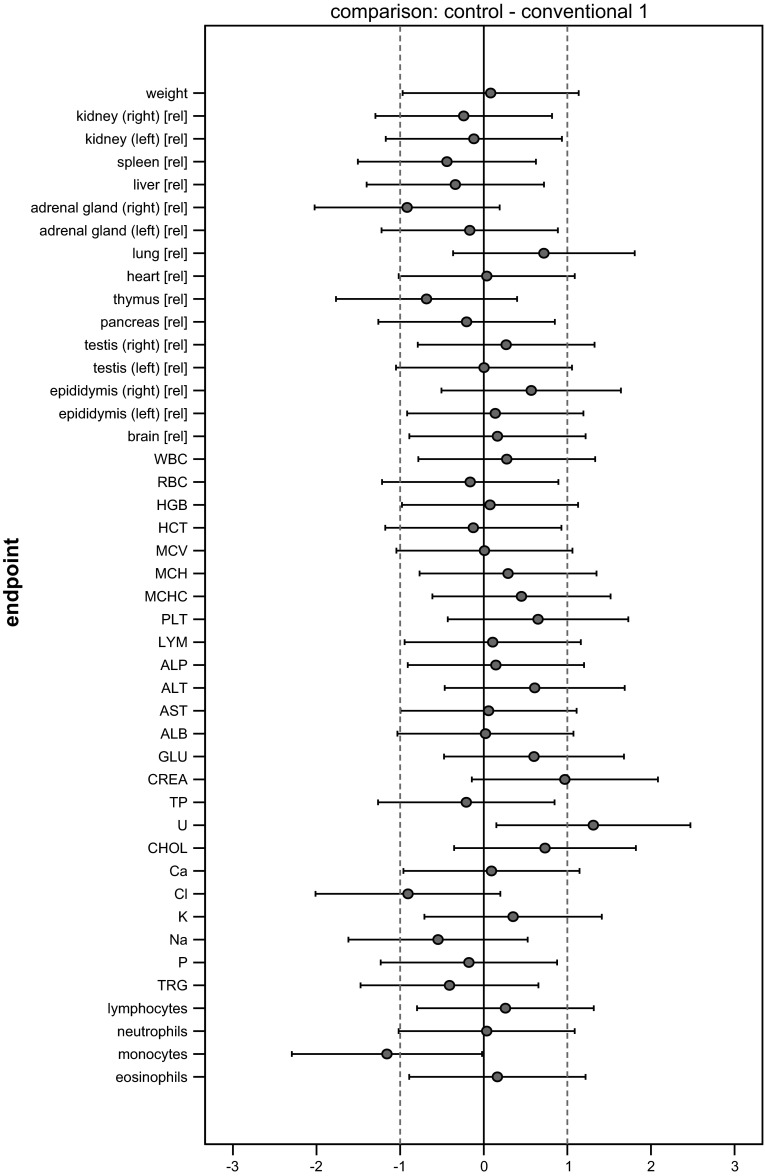

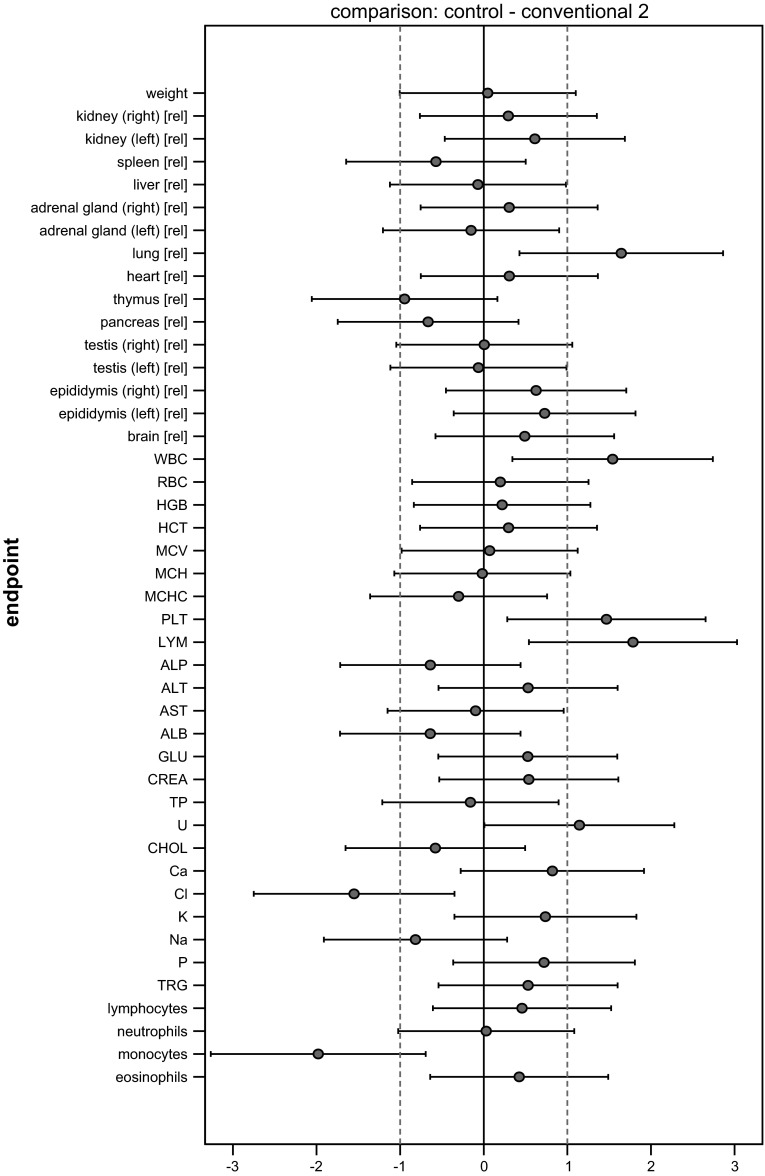
Graphs of standardized effect sizes (**a** control—GMO11 %, **b** control—GMO33 %, **c** control—conventional 1, **d** control—conventional 2) of mean body weight plus all other endpoints, male rats, feeding trial B

The SES of all endpoints (body weight, organ weights, haematology and clinical biochemistry) with their confidence intervals is graphically displayed in Fig. 4. Each graph displays all information extricable from a group comparison. The graphs illustrate the pattern of significances better than the asterisk-marked tables. Moreover, not only the ‘yes’ (= italicized or marked with asterisks)/‘no’ significances but also the sizes of the effects are visualized. Additionally, the biological relevance of the effects (defined here by equivalence limits of ±1.0 SD—dotted lines) can be directly assessed.

The four graphs in Fig. 4 display the four comparisons of interest: control—GMO33 %, control—GMO11 %, control—conventional 1, control—conventional 2. Placing these graphs next to one another allows a direct visual comparison of all comparison patterns. This is the most effective way to assess the outcome of a feeding study at a glance.

## Discussion

The availability of software for fitting LMMs has facilitated their application in biological sciences. Applying linear mixed models to assess developing endpoints like weight allows these data to be analysed in a more comprehensive and consolidated way and facilitates interpretation. First of all, these models enable the complete weight or feed consumption trend to be evaluated and compared, instead of individual points in time. They provide a global statement on group/treatment differences, which is much easier to interpret than a diverse set of single significances between different groups at various points in time of the study. Furthermore, by considering time dependency and averaging over time points, LMMs are more robust against certain deviations from the assumptions on data distribution and therefore model such data more precisely.

The traditional approach (OECD Environment, Health and Safety Publications 2012) applies one-way ANOVA in case of normally distributed variables and equal variation within the treatment groups, and Kruskal–Wallis ANOVA if these assumptions are not met. However, in case of heteroscedasticity also the Kruskal–Wallis test may give inaccurate results; therefore, both approaches are incorrect and will not help. An alternative approach is to apply Welch’s ANOVA test (Kohr and Games 1974), which in turn has been criticized to be unable to handle skewed distributions (Skovlund 2010). Neuhäuser (2010) proposes to apply the generalized Wilcoxon test by Brunner and Munzel (2000). Nevertheless, in simulation studies it has been shown that non-robustness remains a serious problem with all tests, if assumptions of normality and variance homogeneity are not met and a final advice is yet to be given (Skovlund 2010). However—in the absence of an appealing alternative—for our comparison of the LMM and SES approach with the traditional one, we followed the OECD Guidance Document 116 and applied the Kruskal–Wallis test, but it is obvious that the flexible modelling of the variances is a further advantage of the LMM approach.

Reporting and graphically displaying effect sizes and confidence intervals can help to avoid the yes/no decision trap of statistical tests and to illustrate the size of effects in the context of biological relevance. This is supported by several publications in the area of toxicology, particularly by Festing (2014), who demonstrated the use of SES as a data transformation, which can be used in addition to existing techniques to clarify the results of toxicity tests. OECD (2012) states that emphasizing the size of effects and the confidence in them avoids the problem of a small, biologically unimportant effect being declared statistically significant and the artificiality of trying to dichotomize a result into a positive or negative finding on the basis of a *P* value. Furthermore, owing to standardization, all endpoints might be displayed in one graph, allowing a pattern of effects to be assessed instead of single means and significant differences.

In principle, SES might support the assessment of statistical significances with respect to their biological relevance. Since they consider the *effects*, i.e. the differences in endpoints between treatments, they allow an assessment of the toxicological relevance of the sizes of these effects provided that limits for effect sizes of biological/toxicological relevance are also expressed on the same scale. For our study, we followed EFSA (2011) and applied a rough setting of the equivalence limits of ±1.0 SD by assuming that an SES of 1.0 SD or less is unlikely to be of toxicological importance.

There are several issues about standardization that are open to discussion and could be chosen differently. First, the standardization and setting of equivalence limits on a dimensionless scale (as multiples of standard deviation) might be too abstract for interpretation. Toxicologists might prefer to think in the original scales of the various endpoints. Consequently, they might prefer to set equivalence limits or limits of concern individually for each endpoint and each scale. Second, the pooled standard deviation of individual observations SD is determined by both natural variation and measurement uncertainty, and is a priori not expected to be directly related to biological relevance. If external equivalence limits were available, it would be preferable to use these for standardization. Moreover, to assess the relevance of the data of a feeding study, toxicologists compare correlated parameters (like: liver weight, liver necropsy and certain blood values).

The effect size presentation, either supplementing or replacing the traditional *P* value approach, enhances transparency and delivers a more comprehensive overall picture of the information derived from the data, which might support consensus in a decision-making process between all actors involved, namely toxicologists, statisticians and regulators. Furthermore, it helps communicate study results to the public in a more easily understood way.
